# Estrogen accelerates heart regeneration by promoting the inflammatory response in zebrafish

**DOI:** 10.1530/JOE-19-0413

**Published:** 2020-01-24

**Authors:** Shisan Xu, Fangjing Xie, Li Tian, Samane Fallah, Fatemeh Babaei, Sinai H C Manno, Francis A M Manno, Lina Zhu, Kin Fung Wong, Yimin Liang, Rajkumar Ramalingam, Lei Sun, Xin Wang, Robert Plumb, Lee Gethings, Yun Wah Lam, Shuk Han Cheng

**Affiliations:** 1 Department of Biomedical Sciences, College of Veterinary Medicine and Life Science, City University of Hong Kong, Hong Kong SAR, People’s Republic of China; 2 Department of Chemistry, City University of Hong Kong, Hong Kong SAR, People’s Republic of China; 3 School of Biomedical Engineering, Faculty of Engineering, University of Sydney, Sydney, New South Wales, Australia; 4 Department of Biomedical Engineering, Polytechnic University of Hong Kong, Hong Kong SAR, People’s Republic of China; 5 Waters Technologies Corporation, Milford, Massachusetts, USA; 6 State Key Laboratory of Marine Pollution (SKLMP) at City University of Hong Kong, Hong Kong SAR, People’s Republic of China; 7 Department of Materials Science and Engineering, College of Science and Engineering, City University of Hong Kong, Hong Kong SAR, People’s Republic of China

**Keywords:** sexually dimorphic, heart regeneration, estrogen, estrogen receptor, inflammation

## Abstract

Sexual differences have been observed in the onset and prognosis of human cardiovascular diseases, but the underlying mechanisms are not clear. Here, we found that zebrafish heart regeneration is faster in females, can be accelerated by estrogen and is suppressed by the estrogen-antagonist tamoxifen. Injuries to the zebrafish heart, but not other tissues, increased plasma estrogen levels and the expression of estrogen receptors, especially *esr2a*. The resulting endocrine disruption induces the expression of the female-specific protein vitellogenin in male zebrafish. Transcriptomic analyses suggested heart injuries triggered pronounced immune and inflammatory responses in females. These responses, previously shown to elicit heart regeneration, could be enhanced by estrogen treatment in males and reduced by tamoxifen in females. Furthermore, a prior exposure to estrogen preconditioned the zebrafish heart for an accelerated regeneration. Altogether, this study reveals that heart regeneration is modulated by an estrogen-inducible inflammatory response to cardiac injury. These findings elucidate a previously unknown layer of control in zebrafish heart regeneration and provide a new model system for the study of sexual differences in human cardiac repair.

## Introduction

Cardiovascular diseases (CVDs) are the primary cause of death worldwide: killing 17.9 million people in 2015, more than 30% of the global mortality (Roth *et al*. 2017). Gender differences have been reported in the clinical manifestation and recovery of CVD (Ostadal & Ostadal 2014, EUGenMed *et al*. 2015). In various mammalian models of cardiac defects, females consistently demonstrate a lower mortality, less severe disease phenotype and better functional recovery than their male counterparts (Czubryt *et al*. 2006). An understanding of the molecular mechanisms underlying these gender differences will provide an important gateway toward better CVD prevention and treatment. Gene expression profiles of mammalian cardiomyocytes are sexually dimorphic (Isensee *et al*. 2008, Tsuji *et al*. 2017), and estrogen receptor expression is deregulated in some cardiomyopathies (Mahmoodzadeh *et al*. 2006). Despite the apparent involvement of estrogen in CVD, clinical translation of these findings has been lagging, partly due to inconclusive results from large-scale studies on the benefits of post-menopausal hormone replacement in CVD prevention (Low *et al*. 2002). This is attributed, at least in part, to the lack of a mechanistic understanding of the role of estrogen in cardiomyocyte biology, which prevents optimal study design and subject selection in clinical studies (Whayne Jr & Mukherjee 2015).

While adult human cardiomyocytes are virtually unable to re-enter the cell cycle, other vertebrates, including zebrafish, newts and axolotls, demonstrate a remarkable ability to regenerate their hearts (Garcia-Gonzalez & Morrison 2014, Vivien *et al*. 2016). Among these organisms, zebrafish have emerged as one of the most well-established model organisms for the study of heart regeneration. Unlike adult mammals, where myocardial lesions are filled with collagen-rich scars that impair cardiac functions (Hsieh *et al*. 2007, Senyo *et al*. 2013), the scar in zebrafish can be replaced by newly formed cardiomyocytes within 6 months, through extensive cardiomyocytes proliferation, as indicated by the expression of proliferating cell nuclear antigen (PCNA) or phospho-histone H3 (Poss *et al*. 2002, Chablais *et al*. 2011, González-Rosa *et al*. 2011). Additionally, fate mapping experiments have established that proliferating cardiomyocytes originate from mature cardiomyocytes (Jopling *et al*. 2010, Zhang *et al*. 2013). In this regard, the rapid disappearance of the sarcomeric structures and re-expression of embryonic genes, such as embryonic cardiac myosin heavy chain gene (embCMHC), suggest that regeneration largely involves the expansion of dedifferentiated cardiomyocytes (Sallin *et al*. 2015). Interestingly, a recent study has attributed the remarkable regenerative capacity of the zebrafish heart to this organism’s enhanced inflammatory and immune response (Lai *et al*. 2017).

The conservation of genetic pathways between zebrafish and mammals (Howe *et al*. 2013), and the technical and genetic resources offered by the zebrafish research community, make this organism an ideal model for the study of gender biases associated with human CVD. Some observations have suggested a role of sex hormones in zebrafish cardiac development and function. Inhibition of E2 synthesis treatment with aromatase enzyme induces a phenotype similar to congestive heart failure and tamponade in zebrafish embryos (Allgood Jr *et al*. 2013). These conditions are reversed by estradiol (E2) treatment, which has been shown to affect heart rate during zebrafish embryonic development (Romano *et al*. 2017). Despite these findings, the literature in zebrafish heart regeneration is primarily based on studies of a single sex (usually male) and sexual differences in regeneration have never been investigated. In the present study, we examine, for the first time, sexual dimorphism of zebrafish heart regeneration and factors that influence the rate of cardiac regeneration.

## Materials and methods

### Zebrafish maintenance

Zebrafish AB line was acquired from the Zebrafish International Resource Center (ZIRC; Eugene, OR, USA). Fish were maintained in a recirculating system at 28 ± 1°C with a photoperiod of 14 h light/10 h dark as described previously (Xu *et al*. 2018). All the animal procedures used in this study were approved by the Department of Health, Hong Kong, SAR, China (refs (17-18) in DH/HA&P/8/2/5 Pt.1).

### Animal surgery

Adult zebrafish (12- to 18-month-old, the weight is 0.35–0.37 g, with cardiac weight accounting for 0.5% of body weight) were anesthetised by immersion in 0.04% MS-222 (E10521; Sigma-Aldrich) for 3–5 min and then immobilized on a wet sponge. In the sham operation, the heart was exposed but without any further treatment. For ventricular amputation, a portion of the ventricle was excised by using surgical fine scissors (Poss *et al*. 2002). For cryoinjury, the ventricle was touched for 10–12 s with a metal probe pre-chilled in liquid nitrogen (Chablais *et al*. 2011). For fin amputation, the zebrafish caudal fin was cut using a blade after anesthetization (Nachtrab *et al*. 2011). After the operation, fish were placed in a tank of fresh water and revitalization was accelerated by pipetting water onto the gills for a couple of minutes and subsequently. Zebrafish were killed by immersion in overdose concentration of MS-222 at different time points.

### Chemical exposure

**Figure 1:**
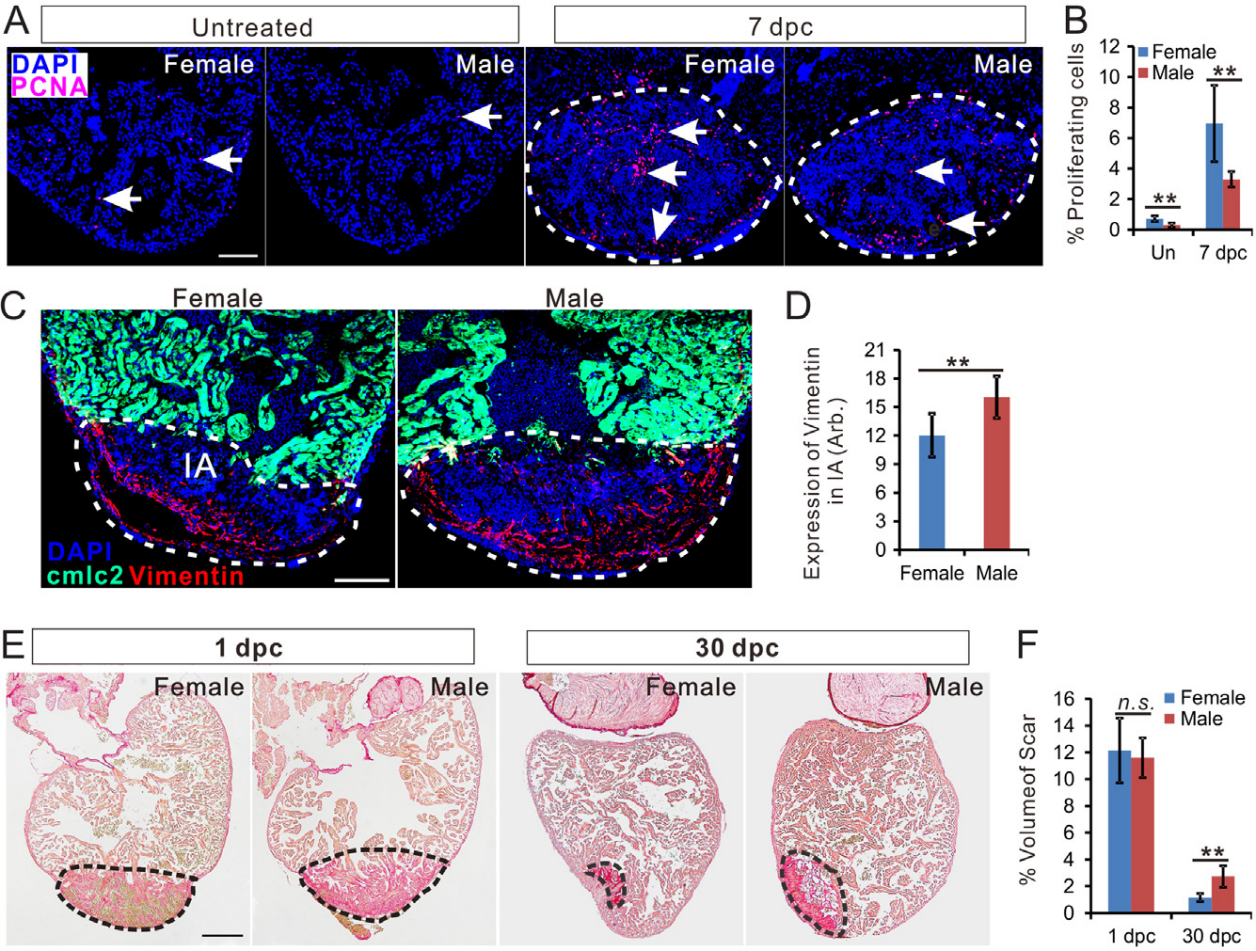
Zebrafish heart regeneration is sexually dimorphic. (A) PCNA immunofluorescence (red) in the heart of untreated female, untreated male, 7 dpc female (c) and 7 dpc male. Scale bars: 100μm. (B) Quantification of percentage of PCNA-positive cells (mean ± s.d., *n* = 7) in panel A. (C) Vimentin immunofluorescence (red) in female and male Tg (*cmlc2*: eGFP, green) zebrafish hearts at 7 dpc. Scale bars: 100 μm. (D) Quantification of vimentin expression in the injured area of female and male zebrafish heart (marked by dashed lines, mean ± s.d., *n* = 8). (E) Picrosirus red staining of female and male zebrafish hearts at 1 dpc and 30 dpc. (F) Quantification of scar volume (marked by dashed lines, mean ± s.d., *n* = 9~12) between females and male zebrafish. Scale bars: 200 μm. Two-tail *t test* in figure B, D and F, **P < 0.01, *n.s.*, not significant. Un, untreated; Dpc, days post-cryoinjury; CI, Cryoinjury.

**Figure 2:**
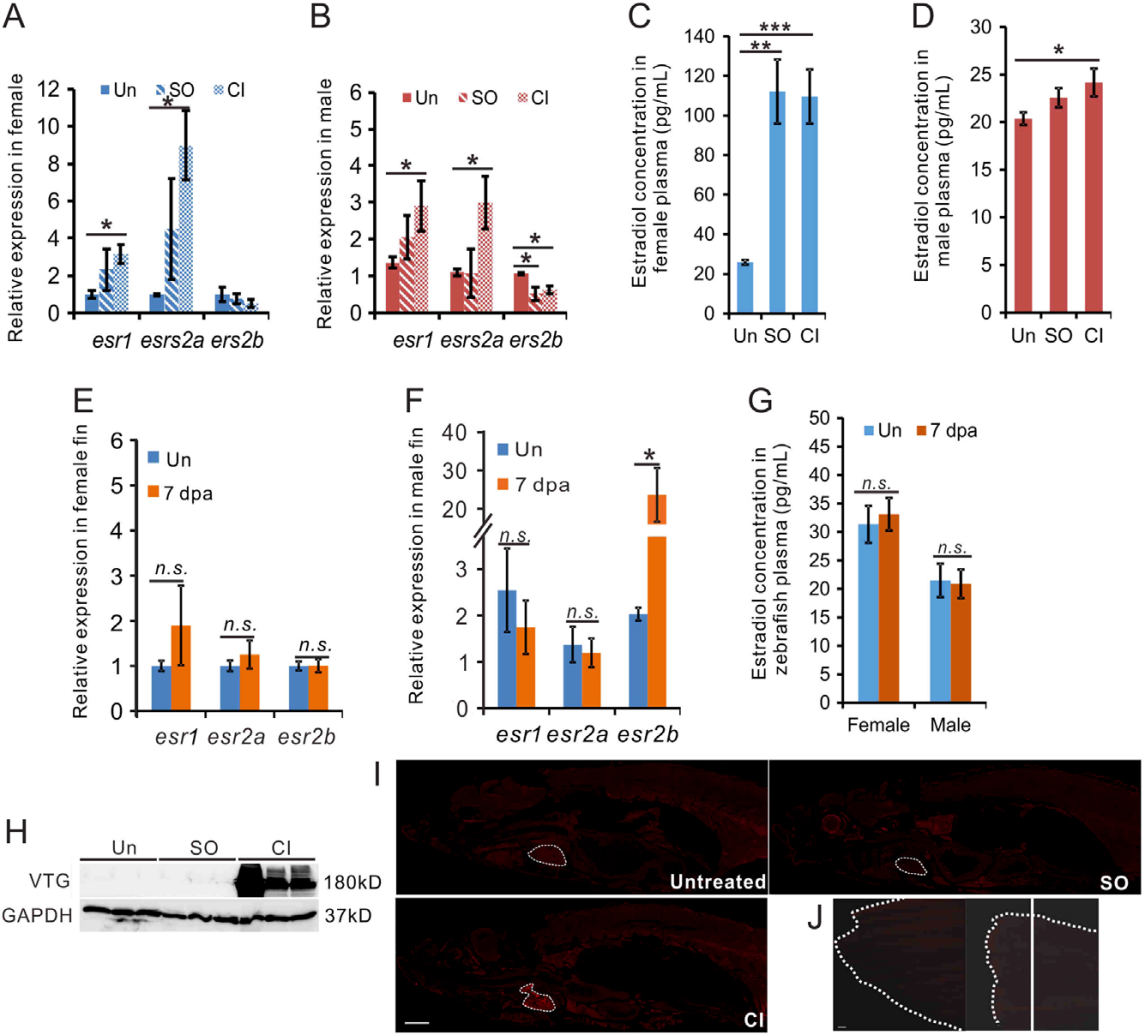
Cardiac damage increased estrogen expression in zebrafish. (A, B, C, D, E, F and G) qRT-PCR showing the expression of estrogen receptor genes in the heart (A, B) and fin (E, F) of zebrafish 7 days after CI; and plasma E2 concentration in zebrafish with heart (C, D) or fin (G) injury at 7 days. *n* = 3, two-tail *t test* (A, B, E, F, G), and one-way ANOVA with LSD *post hoc* test (C, D), *P < 0.05, **P < 0.01, n.s, not significant. (H) Detection of vitellogenin by Western blotting of plasma collected from three independent untreated male zebrafish and fish one day after SO and CI. (I) Detection of VTG (red) in untreated male zebrafish and in male zebrafish on day 7 after heart CI and SO. Scale bars: 1 mm. (J) Detection of VTG in uninjured caudal fin and on 7 days after fin amputation. The dashed white line showed the shape of fin, and the white line showed the amputation site. Scale bar: 1 mm. Un, untreated; SO, sham-operation; and CI, cryoinjury.

**Figure 3:**
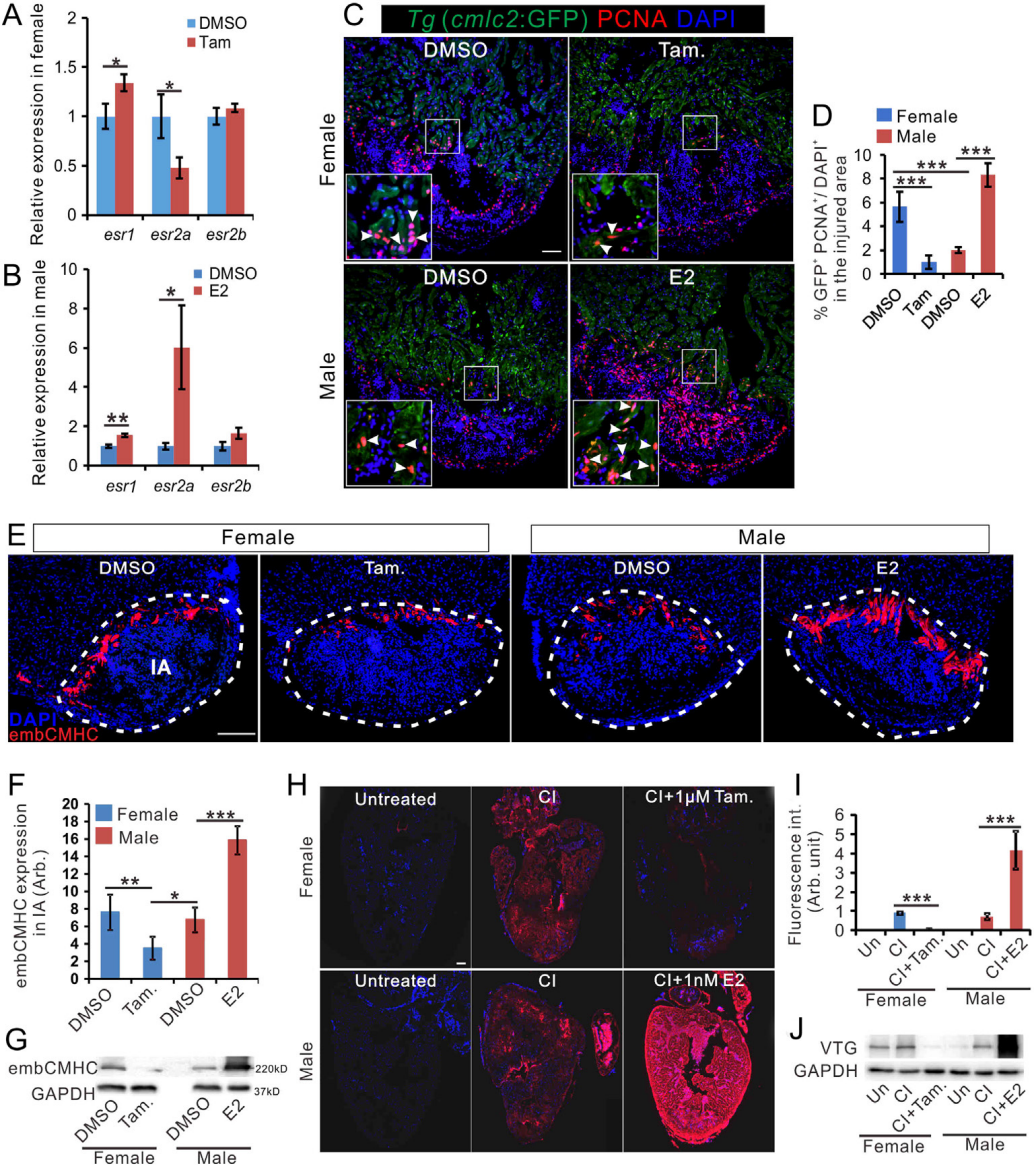
Estrogen promotes regeneration in zebrafish heart. (A and B) qRT-PCR showing the expression of estrogen receptor genes in the heart of female fish (A) and male zebrafish (B) with DMSO, tamoxifen or E2 treatment at 7 days after cryoinjury. *n* = 3. (C) PCNA immunofluorescence (red) in the heart of male *Tg* (*cmlc2*: eGFP) zebrafish exposed to 1 nM E2 for 7 days after CI. Scale bar: 100 μm. (D). Quantification of proliferating cardiomyocytes in panel C (mean ± s.d., *n* = 5). One-way ANOVA with LSD *post hoc* test, *P = 0.05, ***P < 0.001. (E) embCMHC immunofluorescence (red) in the heart of untreated females, females treated with 1 μM tamoxifen (Tam.), untreated males, and males treated with 1 nM E2 for 7 days after CI. Scale bar: 200μm. (F) Quantification of embCMHC staining in panel E (mean ± s.d., *n* = 4~5) in the injured area. One-way ANOVA with LSD *post hoc* test, *P = 0.05, **P = 0.01, ***P < 0.001. (G) Expression of embCMHC in the sample shown in E, as detected by Western blotting. (H) Vitellogenin (VTG) immunofluorescence (red) in the heart of untreated females, 7 dpc females, 7 dpc females treated with 1 μM Tamoxifen, untreated males, 7 dpc males, and 7 dpc males treated with 1nM E2. Scale bar: 100 μm. (I) Quantification of VTG staining (mean ± s.d., *n* = 4~5) in panel H. One-way ANOVA with LSD *post hoc* test, ***P < 0.001. (J) Expression of VTG in samples shown in panel H, as detected by Western blotting. Un, untreated, SO, sham-operation, and CI, cryoinjury.

**Figure 4:**
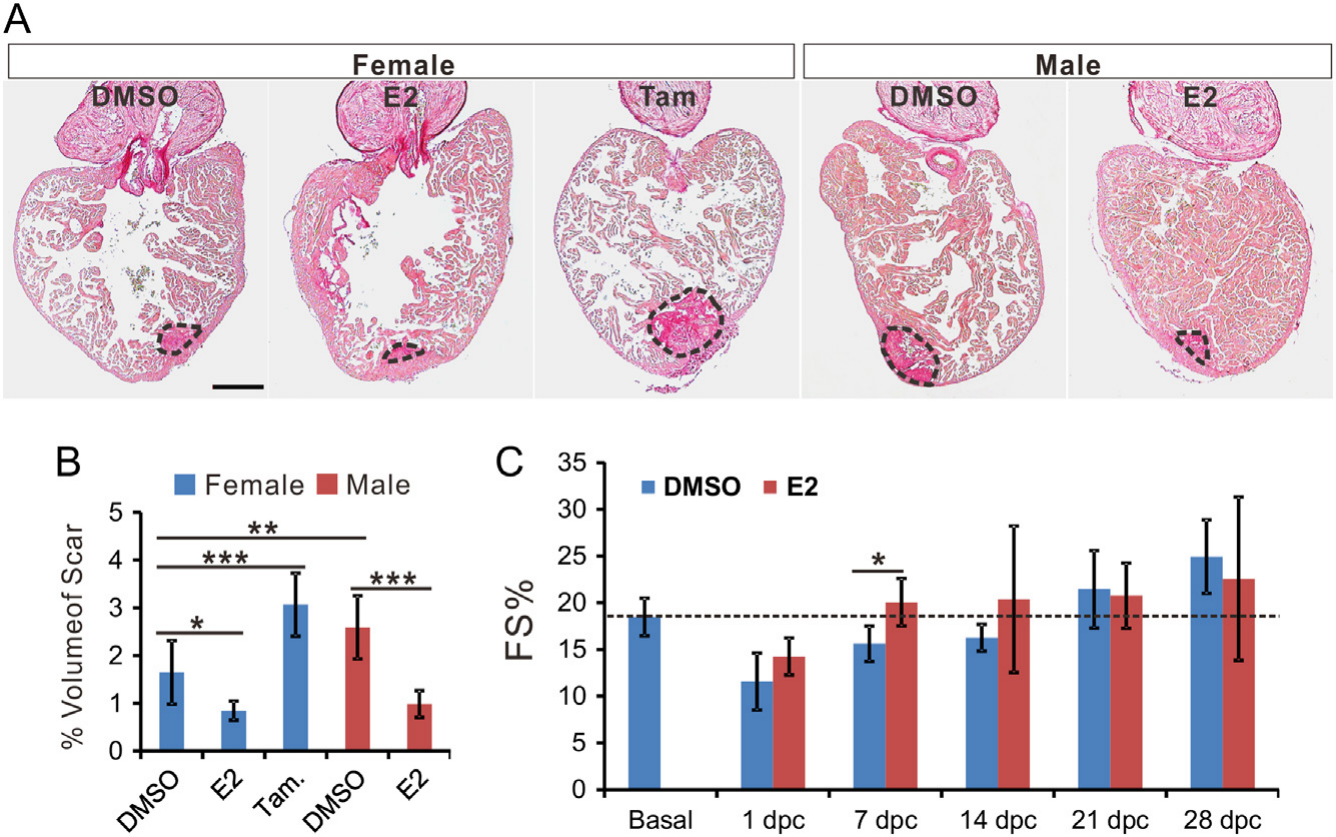
Estrogen accelerates scar reduction and promotes recovery of cardiac function. (A) Picrosirus red staining of the heart from female zebrafish treated with DMSO, E2 (1 nM) and tamoxifen (1 μM); and male zebrafish treated with DMSO and E2 (1 nM) at 30 dpc. Scale bar: 200 μm. (B) Quantification of scar volume from samples in panel A (marked by dash lines, mean ± s.d., *n* = 5~6). One-way ANOVA with LSD *post hoc* test, *P < 0.05, **P < 0.01, and ***P < 0.001. (C) Fractional shortening (FS) measured by echocardiography of male zebrafish before (basal) and at different time points during recovery in DMSO or E2 (1 nM) after CI. Two-way ANOVA with LSD *post hoc* test, *P < 0.05, *n* = 5~6.

**Figure 5:**
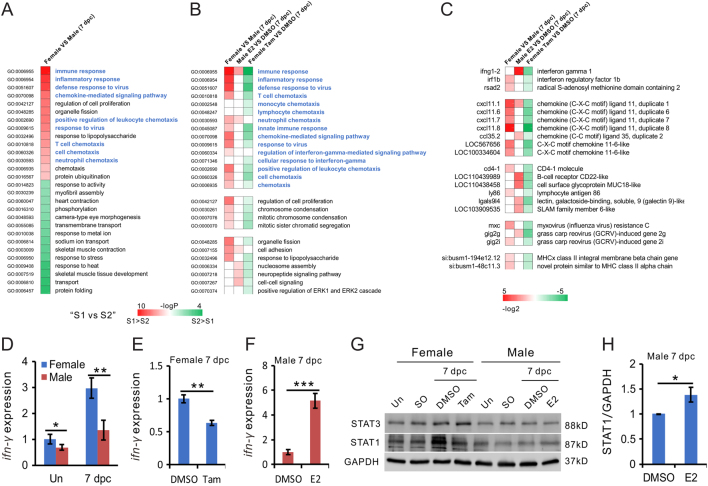
Estrogen induces inflammation in the injured zebrafish heart. A. Gene Ontogeny (GO) terms significantly enriched in genes differentially expressed in female vs male hearts at 7 dpc, *n* = 3. (B) Comparison of gene expression patterns at 7 dpc in the hearts of female vs male, E2 (1 nM)-treated male vs untreated male, and tamoxifen (1 μM)-treated female vs untreated female, *n* = 3. (C) Expression of immune and inflammation-related genes in the datasets, *n* = 3. (D, E and F) Expression of *ifn-γ* at 7dpc in female and male zebrafish heart and uninjured fish (D). Tamoxifen decreased (E) and E2 increased (F) the expression of *ifn-γ* in females and males at 7 dpc. *n* = 4, two-tail *t test*, *P < 0.05, **P < 0.01, ***P < 0.001. (G and H) Western blot of STAT1 and STAT3 in female and male heart at 7 dpc using different treatments. H, bar chart showing the quantification of STAT1 expression in male fish in panel G, *n* = 3, two-tail *t test*, *P < 0.05.

Adult male and female zebrafish, untreated or post surgery, were incubated in water containing 17β-estradiol (E2; E1132, Sigma-Aldrich) at 1 nM, or tamoxifen (T9262, Sigma-Aldrich) at 1 μM, or DMSO (D5879, Sigma-Aldrich), which was used to prepare E2 and tamoxifen, at matching concentrations. The water was changed daily, and the fish were continuously exposed to the vehicle or drugs for durations indicated in Figs 1, 2, 3, 4 and 5.

### Western blotting

Proteins from zebrafish hearts were extracted by using RIPA Lysis and Extraction Buffer (89901, Thermo Scientific) according to manufacturers’ instructions. The BCA Protein Assay kit (23225; Thermo Scientific) was used to determine the total protein concentration of zebrafish plasma and heart protein extract, according to the manufacturer’s instructions. A sample of 20 μg protein per lane was separated in a 10% SDS polyacrylamide gel and then transferred to a PVDF membrane (10600023; GE Healthcare Life Science). The blot was blocked in 5% no fat milk in PBST (0.05% Tween 20 in 1× PBS) for 1 h at room temperature, followed by incubation in primary antibodies diluted in PBST overnight at 4°C. The following primary antibodies were used: mouse anti-GAPDH (60004-1; Proteintech, Rosemont, IL, USA) at 1:10000, mouse anti-zebrafish vitellogenin, JE-2A6 (V01408102; Biosense, Bergen, Norway) at 1:2000, mouse anti-embCMHC (N261.1, DSHB, Iowa City, IA, USA) at 1:500, rabbit anti-STAT1 and rabbit anti-STAT3 (R1408-2 and ET1607-38, both from Huabio, Hangzhou, China) at 1:2000. The following secondary antibodies (all from Millipore) were used: HRP-conjugated rabbit anti-mouse IgG (AP160P) at 1:5000, and HRP-conjugated goat anti-rabbit IgG (AP307P) at 1:5000. The proteins were detected with the EMD Millipore Luminata Western HRP chemiluminescence substrate (WBLUF0500; Millipore) and the signals were visualized with the Western blotting system (C600; Azure Biosystems, Dublin, CA, USA). The band densities were quantified using ImageJ.

### Histology

Organs dissected from zebrafish were fixed with 4% paraformaldehyde at 4°C overnight, dehydrated and embedded in paraffin as previously described (Chablais *et al*. 2011). To measure scar size, the scar volume percentage to the entire ventricle was calculated by summating data from all sections (Xu *et al*. 2018). For the whole mount paraffin sections, the fish were fixed with 2% PFA and 0.05% glutaraldehyde in 80% HistoChoice (H120, Amresco, Cleveland, OH, USA) with 1% sucrose and 1% CaCl_2_ at 4°C for 24 h. The paraffin sections were prepared according to Kong *et al*. (2008).

For immunohistochemistry, antigen retrieval was performed on dewaxed sections in sodium citrate buffer (10 mM sodium citrate, 0.05% Tween 20, pH 6.0) at 95°C for 15 min. The following primary antibodies were used: mouse anti-vimentin (ab8978; Abcam) at 1:200; mouse anti-PCNA (sc-56; Santa Cruz) at 1:200; mouse anti-zebrafish vitellogenin, JE-2A6 at 1:200, mouse anti-embCMHC at 1:50, rabbit polyclonal anti-GFP (ab13970; Abcam) at 1:200. The following secondary antibodies (all from Invitrogen) were used: Cy3-conjugated goat anti-mouse (A10521) or Alexa Fluor 488-conjugated goat anti-rabbit (A11034) antibodies at 1:500. The sections were mounted with a cover slide in 50% glycerol with PBS and images were acquired using an Olympus BX61 microscope.

### Plasma E2 concentration measurement

Plasma from three individual females or males were pooled, and three biological replicates were performed (Babaei *et al*. 2013). The concentration of E2 in plasma was measured using Estradiol ELISA Kit (582251, Cayman).

### Quantitative real-time PCR (qRT-PCR) and RNA sequencing

Total RNA was extracted from zebrafish heart and fin using NucleoZOL reagent (740404; MACHEREY-NAGEL, Düren, Germany). Three fish were pooled together as one biological replicate, and three replications were performed. A sample of 1 μg total RNA was decontaminated using RQ1 RNA-free DNase (M6101; Promega) and then cDNA was synthesized using the PrimeScript RT reagent kit (6210B; Takara) according to the manufacturers’ instructions. The expression of each gene was determined by qRT-PCR using the SYBR Premix Ex Taq (RR402A; Takara), and *β-actin* was used as the reference gene. The qRT-PCR analysis was performed in triplicate for each gene, and the results were analysed using the 2^ΔΔCT^ method. All levels of gene expression were normalized to *β-actin* and fold change was calculated by setting the control females to 1. The primer sequences are list in Table 1.

**Table 1:** Primer sequence for qRT-PCR.

Gene	Sequence
esr F	CAGGACCAGCCCGATTCC
esr R	TTAGGGTACATGGGTGAGAGTTTG
esr2a F	CTCACAGCACGGACCCTAAAC
esr2a R	GGTTGTCCATCCTCCCGAAAC
esr2b F	CGCTCGGCATGGACAAC
esr2b R	CCCATGCGGTGGAGAGTAAT
ifn-γ F	CTATGGGCGATCAAGGAAAA
ifn-γ R	CTTTAGCCTGCCGTCTCTTG
β-actin F	GCTGACAGGATGCAGAAGGA
β-actin R	TAGAAGCATTTGCGGTGGAC
vtg1 F	ACTACCAACTGGCTGCTTAC
vtg1 R	ACCATCGGCACAGATCTTC
vtg2 F	GGTGACTGGAAGATCCAAG
vtg2 R	TCATGCGGCATTGGCTGG
vtg3 F	CAGATGGCTTTATCGGCGTGAC
vtg3 R	CACGGCAGGCCCATTGAAAC
vtg4 F	TCACTGTTCCCATCAATCCA
vtg4 R	TACAAACATCTCAACAATTAGCA
vtg5 F	GATTCCAGAGATCACAATGTCA
vtg5 R	CAATTAAACATTCATCACACATG
vtg6 F	GGATTCACAAGTATATTAAGGAGG
vtg6 R	ACACTTGCAGGGTATTTATTAGC
vtg7 F	ATTCCTCTGCCAGTTGCTGT
vtg7 R	ACTTGCAGAGAGGACGTTTATT

For RNA sequencing, total RNA was extracted and decontaminated, and sequencing was performed using the BGISEQ-500 platform, generating an average of 21.83 M reads per sample. The reference genome can be accessed at: http://www.ncbi.nlm.nih.gov/genome/50?genome_assembly_id=210873. The sequencing and primary analysis were performed by BGI (Shenzhen, China), three replicates of each sample were performed. Comparative GSEA (Gene set enrichment analysis) was performed and union enrichment maps were constructed using the R package HTSanalyzeR2 (https://github.com/CityUHK-CompBio/HTSanalyzeR2). The purpose here was on biological processes using Gene Ontology (GO). A 1000 folds permutation was employed to derive statistical significance with an adjusted *P* value <0.05.

### Echocardiography

For echocardiography, zebrafish were anaesthetized with 0.02% MS-222 and fixed on a sponge in the supine position. The echocardiography was performed using the Vevo LAZR Multi-modality Imaging Platform (FUJIFILM VisualSonics) under B-mode (50 MHz, 77 fps) at 20°C as previously described (González-Rosa *et al*. 2014, Hein *et al*. 2015, Wang *et al*. 2017). Ejection fraction (EF, which indicates the volumetric output of blood from the heart) and fractional shortening (FS, which indicates the systolic function of the heart) were acquired using a plug-in of Vevo LAB (Vevo Strain) under B-mode images. The window was set to be 300 frames long, starting with diastalsis.

### Quantification and statistical analysis

**Figure 6:**
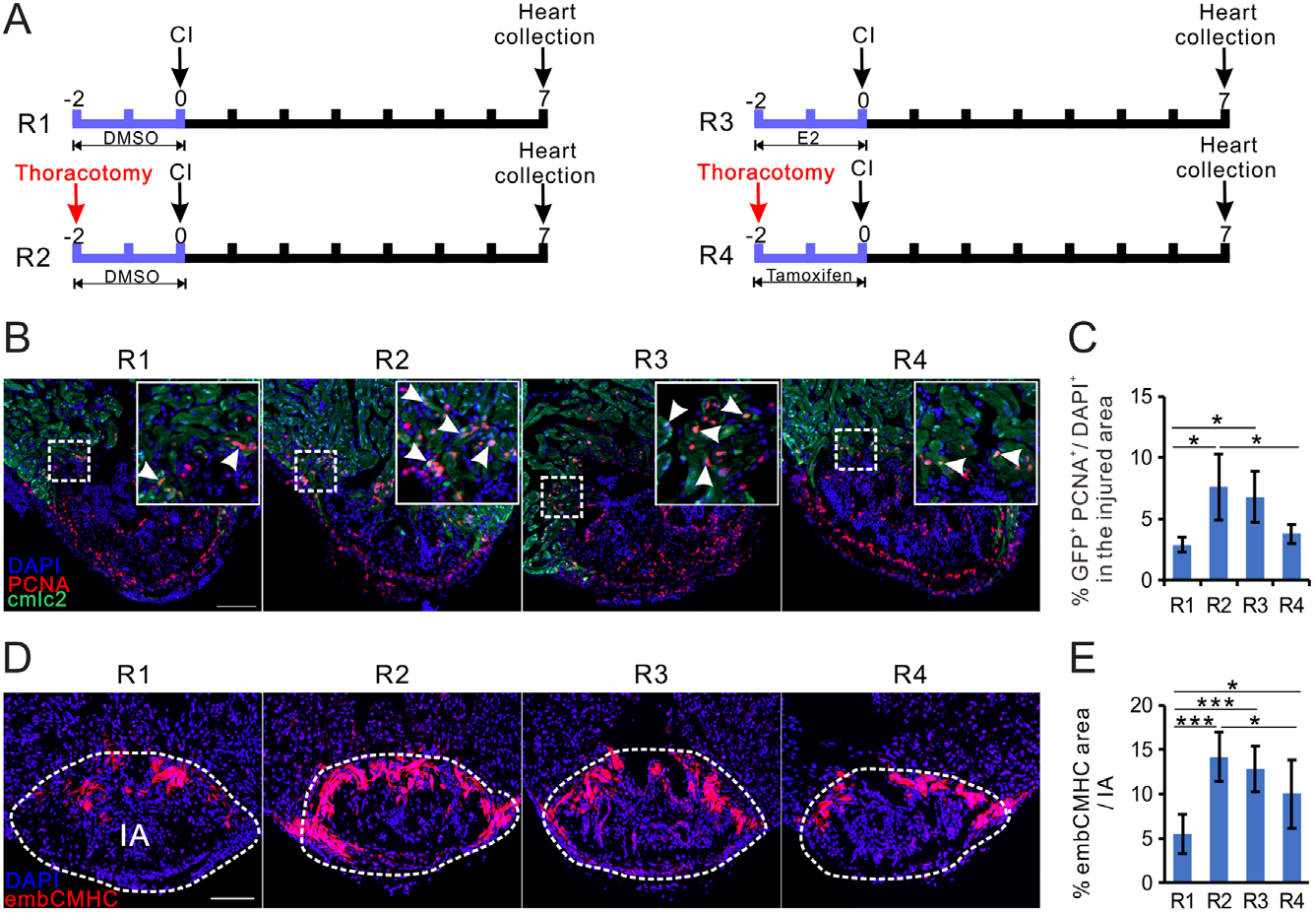
Estrogen preconditioning treatment promotes zebrafish heart regenerative process. (A) Experimental design of data presented in this figure. (B) PCNA immunofluorescence (red) in the heart of male *Tg* (*cmlc2*: eGFP) zebrafish heart from regimens R1-R4. Arrowheads in the white line bounded area indicate proliferating cardiomyocytes (PCNA^+^/GFP^+^). Scale bar: 100 μm. (C) Quantification of proliferating cardiomyocytes (PCNA^+^/GFP^+^) in panel B (mean ± s.d., *n* = 4~5). One-way ANOVA with LSD *post hoc* test, *P < 0.05. (D) embCMHC immunofluorescence (red) in the male heart from regimens R1-R4. (E) Quantification of embCMHC staining in panel E (mean ± s.d., *n* = 5~6) in the injured area. Scale bars: 100 μm. One-way ANOVA with LSD *post hoc* test, *P = 0.05, ***P < 0.001.

Three different images were taken for each heart to use for immunofluorescent quantification. The percentages of proliferating cells in the ventricle or injured area was calculated as the ratio of PCNA positive cells/DAPI. For untreated fish, all nuclei in the whole ventricle were counted; for the cryoinjured zebrafish, only the nuclei in the injured area were counted (marked by a white dash line in Fig. 1). The area of vimentin and embCMHC expression were quantified in the injured area using ImageJ. The percentage of the expression with respect to the area of the injured area was calculated as previously described (de Preux Charles *et al*. 2016*b*). For quantification of proliferating cardiomyocytes, the PCNA^+^/GPF^+^/double positive cardiomyocytes within 100 μm of the vicinity of the injured area were calculated, and normalized with the injured area as seen in Figs 3C and 6B. The data were expressed as the mean ± s.d.. Statistical analysis was performed using Student’s two-tailed *t*-test and a one-way ANOVA. In some experiments (as indicated in Figure legends, a two-way ANOVA with LSD* post hoc* test was used.

## Results

### Zebrafish heart regeneration is sexually dimorphic

Female and male zebrafish, matched for age and weight, were subjected to cardiac damage by cryoinjury. On day 7 post cryoinjury (7 dpc), female hearts contained a significantly higher number of PCNA-positive cells (Fig. 1A) and less vimentin immunoreactivity (Fig. 1C) compared to male zebrafish (Fig. 1B and D). These results indicate more cell proliferation and fewer scar-forming fibroblasts in the regenerating female heart. Uninjured female hearts contained a significantly higher number of PCNA-positive cells compared to male hearts (Fig. 1A and B), indicating a higher baseline proliferative activity in female cardiomyocytes. We compared the relative scar volume in female and male hearts and showed that while scar volume was similar in both sexes at 1 dpc, scar volume in male fish was about two-folds larger than in female fish at 30 dpc (Fig. 1E and F). Together, these data support sexual dimorphism of zebrafish heart regeneration, with females regenerating their hearts faster than males.

### Endocrine disruption of zebrafish following cardiac injury

Our observations on the sexual dimorphism of zebrafish heart regeneration prompted us to examine the involvement of estrogen in this process. toward this aim, the expression of all three nuclear ERs (Lu *et al*. 2017) was examined in zebrafish heart after cryoinjury or sham operation, as well as in the heart of uninjured fish (untreated). A sham operation was performed by identical procedures that preceded in cryoinjury, including the opening of thoracic cavity and exposing the heart. At 7 days post surgery, the expression of* esr1* and *esr2a* was significantly induced in female hearts (Fig. 2A). Interestingly, the expression of these two ERs in male hearts was also significantly increased, though their increase was not as pronounced as in the female heart (Fig. 2B). Sham operation induced the expression level of *esr2a* (in female) and *esr1* (in both sexes), but to a lesser extent compared to the effect of cryoinjury. *Esr2b* was suppressed by both cryoinjury and sham operation in either sex.

We asked whether the E2 level in fish was affected after heart injury, as ERs are estrogen inducible genes. As expected, the plasma E2 level in untreated female fish (~25.8 pg/mL) was slightly higher than in males (~20.4 pg/mL) (Fig. 2C and D). Consistent with the upregulation of *esr1* and *esr2a*, the plasma E2 level was increased ~5-fold in both sham-operated and cryoinjured female fish (Fig. 2C). Plasma E2 in male fish was increased by 20 and 10% after cryoinjury and sham-operation, respectively (Fig. 2D). However, the expression of *esr1* and *esr2a* in the fin was not significantly altered after amputation in either female or male fish, though the expression of *esr2b* was significantly increased after amputation (Fig. 2E and F). Further, plasma E2 levels after caudal fin amputation were virtually unchanged as compared to untreated controls (Fig. 2G). Overall, these data suggest that injury to the heart, but not other tissues, induces estrogen secretion and response in zebrafish.

To test the biochemical consequence of this hormonal change, we examined the sexual dimorphism in zebrafish plasma proteins (Babaei *et al*. 2013, Li *et al*. 2016). Quantitative proteomic analysis was used to study the relative abundance of 18 known sexually dimorphic plasma proteins in plasma collected from untreated, sham-operated and ventricular amputated male zebrafish (Supplementary Fig. 1A and Supplementary Table 1, see section on supplementary materials given at the end of this article). Most female-biased plasma proteins increased after cardiac damage, as compared to sham operation. Conversely, all but two male-biased plasma proteins were downregulated after ventricular injury. These data indicate that although the impact of heart injury on estrogen levels in males was relatively minor, it was sufficient enough to shift the gender characteristic of the male zebrafish plasma toward a more feminised phenotype.

Western blotting detected vitellogenin in the plasma of male zebrafish as early as day 1 after both cryoinjury (Fig. 2H) compared to sham or uninjured fish. Our plasma proteomic analysis indicated the presence of vitellogenin isoforms in the plasma of male zebrafish after heart injury (Supplementary Fig. 1B). Vitellogenin is specifically expressed in females and is routinely used as a biomarker for endocrine disruption in males (Scott & Robinson 2008). Vitellogenin has been reported to be an acute phase response protein, overexpressed within hours after LPS stimulation (Tong *et al*. 2010). However, the abundance of vitellogenin in the plasma was much higher in heart-damaged fish than in sham-operated fish, even though the latter also suffered from extensive tissue damage. Moreover, plasma proteomics showed that other known acute phase response proteins (Roy *et al*. 2017), such as the complement and coagulating factors, were downregulated in the regenerating fish heart compared to the sham-operated fish (Supplementary Fig. 1B and Supplementary Table 2). This suggests that the presence of vitellogenin in male plasma after heart injury is not a consequence of the injury-related infection, but is specifically associated with heart regeneration.

In female fish, vitellogenin is synthesised in the liver and transported via plasma to the oocytes (Hara *et al*. 2016). To test whether the overexpression of vitellogenin in male fish could be traced back to liver, we measured the mRNA levels of seven known vitellogenin isoforms (Wang *et al*. 2005) in zebrafish after SO or CI (Supplementary Fig. 3). We observed that both treatments led to a significant increase of all vitellogenin isoforms, especially VTG5. The increase of vitellogenin expression at RNA level in the liver of SO fish was not consistent with our immunofluorescence observations, suggesting that post-transcriptional mechanisms may be involved in regulating VTG protein levels in heart regeneration.

We then examined the tissue distribution of vitellogenin in male zebrafish during heart regeneration. Whole mount immunohistochemistry showed that vitellogenin accumulated in the injured heart of male fish (Fig. 2I) and was not observed in the proximity of the chest wound of sham-operated fish. This indicates that vitellogenin accumulation in male fish is specifically associated with cardiac damage and is not related to general wound healing. Moreover, vitellogenin was not detectable in the male caudal fin on day 7 after amputation (Fig. 2J), confirming that vitellogenin accumulation is not a general consequence of tissue regeneration or repair, but more pertinent to cardiac injury. The individual tissue immunohistochemistry analysis confirmed that vitellogenin was detected in the heart, but not in the gill, kidney and liver (Supplementary Fig. 1C). Vitellogenin was observed in the entire regenerating heart, not restricted to the wound.

### Estrogen promotes heart regeneration in zebrafish

What is the effect of estrogen on heart regeneration? At 7 dpc, tamoxifen inhibited the expression of *esr2a* in the female heart (Fig. 3A), while E2 increased the expression of *esr2a* in the male heart (Fig. 3B). Male zebrafish exposed to E2 after cryoinjury displayed a ~4-fold increase in cardiomyocyte proliferation in the vicinity of the injured area (Fig. 3C and D). Additionally, male fish treated with E2 increased cardiomyocyte dedifferentiation, as judged by the expression of embCMHC (Fig. 3E, F and G). Conversely, treatment of female fish with the estrogen receptor antagonist tamoxifen resulted in a ~4-fold decrease in cardiomyocyte proliferation in the vicinity of injured area (Fig. 3A) and ~2-fold decrease in embCMHC expression (Fig. 3E, F and G). Furthermore, E2 treatment of male fish increased the level of vitellogenin in regenerating hearts, while tamoxifen treatment of female fish reduced vitellogenin accumulation (Fig. 3H, I and J).

We then investigated whether tamoxifen and estrogen affected scar degradation after heart injury. Tamoxifen significantly increased scar volume in female fish (Fig. 4A and B), whereas E2 quickened scar degradation in both female and male fish. Moreover, echocardiography, which allows the non-invasive monitoring of cardiac performance of the same fish during regeneration (Wang *et al*. 2017), indicated that E2 treatment of male fish after cryoinjury accelerated the restoration of fractional shortening (FS) time (but not EF, data not show) to the pre-injury level (Fig. 4C). FS is a parameter that indicates the cardiac contractile force and has previously been shown to correlate with zebrafish cardiac recovery after cryoinjury (Hein *et al*. 2015). Here, the restoration of FS confirms the role of E2 in promoting the recovery of physiological function after cardiac damage in male zebrafish. Overall, our data show that estrogen promoted the regeneration and the recovery of heart function.

### Estrogen enhances immune and inflammatory responses in regenerating heart

To elucidate the mechanism underlying the sexual difference in zebrafish heart regeneration, we performed comparative transcriptomic analyses on RNA extracted from female and male hearts at 7 dpc. A total of 1050 genes were found to be differentially expressed (more than two-fold) and statistically significant across the three replicates between the two sexes (Supplementary File 3). Gene ontology (GO) analyses revealed that most of the biological processes enriched for the female-biased genes were related to immunological functions, such as immune response, inflammatory response and chemotaxis for immune cells. On the other hand, male-biased genes were more diverse in function, such as protein homeostasis, stress response and muscle contraction (Fig. 5A and Supplementary File 4). Gene set enrichment analysis (GSEA) (Subramanian *et al*. 2005) confirmed that immune-related pathways were among the most sexually dimorphic in post-injured zebrafish hearts (Supplementary Fig. 2).

To test how much the observed female-specific gene expression pattern in heart regeneration was shaped by estrogen, the respective effects of E2 treatment of male fish, and tamoxifen treatment of female fish, on the transcriptome of injured hearts were investigated. Figure 5B (Supplementary File 5) shows that tamoxifen treatment reversed female-specific gene expression signatures in the injured heart, whereas genes involved in immune response and neutrophil chemotaxis were upregulated in estrogen-treated males. Among the genes whose expression level in injured hearts were sexually dimorphic, and/or reciprocally regulated by E2 and tamoxifen, were interferon-gamma (*ifn-γ*), interferon regulatory factor 1b (*irf1b*) and various isoforms of *cxcl11* (Fig. 5C and Supplementary File 6). In mammals, both *irf1* and *cxcl11* are *ifn-γ*-inducible (Yang *et al*. 2007, Flodström & Eizirik 1997), suggesting the interferon-gamma pathway maybe instrumental to the sexual dimorphism in heart regeneration. The qRT-PCR analysis confirmed that the expression level of *ifn-γ* was higher in female than male in both uninjured and 7 dpc heart, though its expression was increased in the female and male heart after injury (Fig. 5D). However, the *ifn-γ* expression after heart injury was significantly stimulated by E2 treatment in male zebrafish and suppressed by tamoxifen treatment in female zebrafish (Fig. 5E and F), consistent with the role of estrogen in *ifn-γ* expression in other tissues (Fox *et al*. 1991, Hao *et al*. 2013).

Consistent with the female-specific induction of *ifn-γ* expression, a significant increase in STAT1, whose expression is known to be upregulated by IFN*-γ* in mammals (Horvath 2004), was observed in regenerating female hearts, but not in males (Fig. 5G). The upregulation of STAT1 in female hearts could be reversed by tamoxifen (Fig. 5G). In males, E2 treatment resulted in a moderate, but statistically significant increase in STAT1 protein in regenerating hearts (Fig. 5G and H). These results are consistent with the recent observation that STAT1 is an estrogen-responsive gene in mammals (Young *et al*. 2017). Interestingly, it is known that the STAT3 gene is essential to injury-induced cardiomyocyte proliferation (Fang *et al*. 2013). Our data showed that STAT3 was also induced in female hearts after injury, but this induction was not tamoxifen-sensitive (Fig. 5G), suggesting STAT1 may be more important than STAT3 in orchestrating sexual differences in regeneration rates.

### Early estrogen preconditioning sensitizes the zebrafish heart for regeneration

Sham operation preconditioned zebrafish showed more efficient regeneration if their hearts were damaged shortly afterwards, possibly due to an increase in inflammation as a result of thoracotomy (de Preux Charles *et al*. 2016*a*,*b*). We observed that sham operation led to a significant increase in the expression of estrogen receptors (Fig. 2A and B) and plasma E2 (Fig. 2C and D) at 7 dpc. To test whether estrogen is instrumental to the preconditioning effect, we treated thoracotomised male zebrafish with tamoxifen and normal male zebrafish with E2 for 2 days prior to cryoinjury (Fig. 6A). As expected, regeneration, as judged by the number of proliferating cardiomyocytes and the expression of embCMHC, were enhanced by thoracotomy (Fig. 6B, C and D), confirming the occurrence of the preconditioning effect. However, this effect was significantly reduced when the fish were exposed to tamoxifen during the preconditioning period (Fig. 6B, C and D). Furthermore, male zebrafish pre-treated with E2 for 2 days prior to cryoinjury showed a significant increase in proliferating cardiomyocytes and embCMHC expression, when compared to DMSO pre-treated fish. Hence, E2 pre-treatment is effective in inducing the preconditioning effect on heart regeneration.

## Discussion

In this study, we demonstrated by cellular, anatomical, physiological and biochemical methods (Figs 1 and 2) that zebrafish heart regeneration is sexually dimorphic. To our knowledge, this is the first evidence of sexual dimorphism in heart regeneration. Sexual differences in regeneration of other tissues have been observed in mammals, but have not been documented for the heart (Harada *et al*. 2003, Deasy *et al*. 2007). In zebrafish, the pectoral fin is the only tissue that has been shown to demonstrate a sexually dimorphic regenerative capacity (Nachtrab *et al*. 2011). Like their hearts, males regenerate the pectoral fin more slowly and often incompletely. However, this phenomenon was not observed as robustly in other fins of the zebrafish (Nachtrab *et al*. 2011), suggesting that this sexual dimorphism is related to the unique role of pectoral fins in reproduction (Kang *et al*. 2013).

We demonstrated the positive effect of estrogen on heart regeneration: E2 accelerated heart regeneration in males (Figs 3 and 4), and tamoxifen retarded heart regeneration in females (Figs 3 and 4). Our gene expression analyses suggested that female hearts demonstrated a stronger immune and inflammatory responses to cryoinjury. In particular, the expression of *ifn-γ*, as well as *ifn-γ*-inducible factors, in regenerating hearts was highly sexually dimorphic and E2 sensitive (Fig. 5). As inflammation is essential for tissue regeneration (Eming *et al*. 2017), including cardiac repair, our observation is consistent with the higher heart regeneration rate in female zebrafish. Importantly, a comparison between fish species with different heart regeneration rates, reveals that the high heart regeneration capacity of zebrafish is likely due to its enhanced inflammatory response to heart injury (Lai *et al*. 2017). These authors also discovered that treatment with poly I:C, a viral mimic known to stimulate *ifn-γ*-responsive genes (Farina *et al*. 2010), can enable heart regeneration in medaka, a species normally unable to repair heart injuries. The immune-promoting effects of estrogen have been well documented in fish and mammals (Burgos-Aceves *et al*. 2016, Taneja 2018). In addition, sham operation preconditioning induced inflammation and promoted heart regenerative program (de Preux Charles *et al*. 2016*b*). Interestingly, our data showed that sham-operation was sufficient in increasing the plasma level of E2 in zebrafish (Fig. 2C and D) and that this preconditioning effect is E2 dependent (Fig. 6).

**Figure 7:**
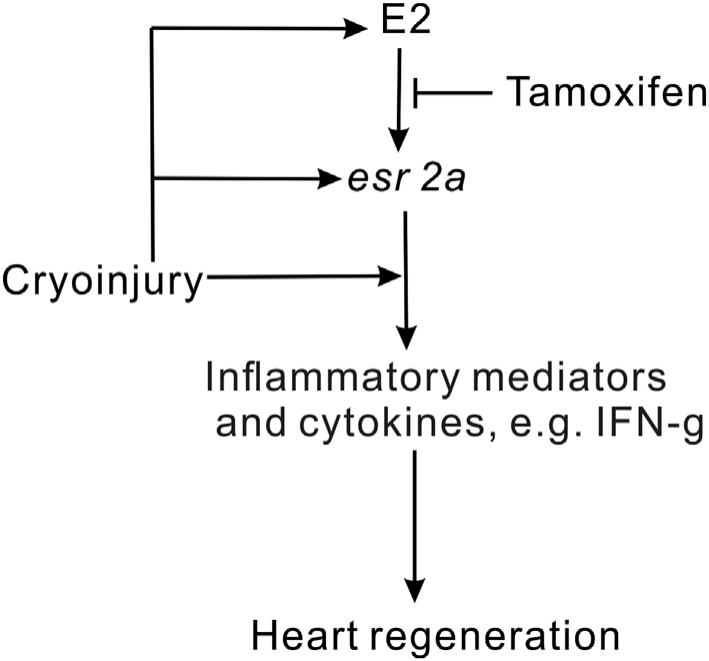
Proposed model estrogen role in zebrafish heart regeneration. Heart cryoinjury triggers the inflammation response and plasma estrogen upregulation. The increase of plasma estrogen induces expression of *esr2a*, which can be inhibited by tamoxifen. Estrogen promotes heart regeneration through *esr2a* induced and injury directly induced inflammation response.

We have revealed a previously unknown aspect of tissue regeneration in zebrafish: endocrine disruption induces the expression of female-specific proteins in males (i.e. vitellogenin). Our data reveal that cardiac damage triggers the expression of estrogen receptors, notably *esr2a* (Fig. 2A and B) and secretion of E2 (Fig. 2C and D), in both sexes. Interestingly, a genetic variant of *esr2* has been identified as a risk factor of myocardial infraction (Domingues-Montanari *et al*. 2008). Additionally, *ERbeta*, a mammalian homologue of *esr2a*, has recently been found to locate in mitochondria and play bioenergetic roles (Liao *et al*. 2015). In this regard, a ~9-fold increase in *esr2a* expression in female hearts was observed at 7 dpc (Fig. 2A). Such a dramatic increase in ER expression, compounded by the ~5-fold increase of plasma E2 levels (Fig. 2C), likely sensitises estrogen-responsive genes and amplifies the estrogen-dependent inflammatory response of the female heart. We postulate this can further enhance heart regeneration in females and establish the sexual dimorphism of this process (Fig. 7). The stimulation of estrogen levels and receptors by heart injury also operates in male fish, albeit to a lesser extent. Interestingly, the level of serum estrogen among acute myocardial infarction and unstable angina patients was found to be significantly higher than in the normal group and intensive care patients (AKSUT *et al*. 1986), suggesting that cardiac lesions, but not other traumas, can lead to an increase in estrogen level in humans. We now show that the unique role of estrogen in heart repair is evolutionarily conserved. For example, endocrine disruption was not associated with zebrafish fin regeneration (this study, Fig. 2J) and estrogen does not affect the rate of fin regeneration in zebrafish larvae (Sun *et al*. 2019).

It is interesting to examine how the systemic increase of estrogen as a result of heart injury may impact the function of other organs during heart regeneration. One of the consequences of endocrine disruption is the detection of vitellogenin in males, an occurrence generally regarded as the hallmark of endocrine disruption by environmental agents (Scott & Robinson 2008). The accumulation of vitellogenin in regenerating hearts, but not in other tissues (Fig. 2 and Supplementary Fig. 2C), suggests a functional role in cardiac regeneration, rather than as a collateral consequence of estrogen secretion. We did not detect a significant level of vitellogenin transcripts in regenerating hearts of either sex (data not shown). Our data reveal the synthesis of vitellogenin, especially VTG5, in the male liver after cardiac damage (Supplementary Fig. 3) and its presence in plasma (Fig. 2H and Supplementary Fig. 1B), suggesting that vitellogenin was delivered and accumulated in the damaged hearts. The canonical function of vitellogenin is to transport lipids, calcium and phosphate to developing oocytes (Arukwe & Goksøyr 2003, Hara *et al*. 2016). We hypothesise that vitellogenin, especially VTG5, might play a similar role in the regenerating heart. Future investigations will include the identification of the target cell types of vitellogenin in the regenerating heart and the characterisation of vitellogenin cargo(s) delivered to these cells.

As a major physiological challenge, tissue regeneration likely requires the coordinated contributions from multiple tissue systems. This study reveals, for the first time, that the involvement of the endocrine and immune systems in zebrafish heart regeneration and establish the use of zebrafish as a model for the study of sexual differences in human cardiac pathology.

## Supplementary Material

Figure S1. Vitellogenin accumulates in the male zebrafish heart after cardiac damage. A. Ratio of the relative abundance of selected plasma proteins on days 1, 3 and 7 post cardiac injury. VA: ventricular amputation, SO: sham operation. N=3-5. B. Relative abundance of known acute response proteins in zebrafish plasma collected on day 1 (D1), day 3 (D3) and day 7 (D7) after ventricular amputation (VA) and sham operation (SO). Colours represent the log2 ratio of protein abundance in VA vs SO. N=3-5, only data with p<0.06 are shown. C. Vitellogenin (VTG) immunofluorescence (green) in male zebrafish heart, gill, kidney and liver on day 5 after VA. Scale bar: 200um.

Figure S2. Comparative transcriptome analyses between female and male zebrafish 7 days post cardiac injury. A. Enrichment map showing the gene profile in female and male heart at 7 dpc. Red color represents NES>0, and gene set upregulated; blue color represents NES< 0, and gene set downregulated. The strength of the red or blue color represents the value of the adjusted p-value. B. GSEA plot showing the location of the maximum enrichment score (ES) and the leading-edge subset of immune response genes.

Figure S3. Expression of vitellogenin isoforms in the male liver are stimulated by cardiac damage. Expression levels of vtg 1-7 in male zebrafish liver with heart untreated (Un), sham operation (SO) and cryoinjury at 7 dpc, as measured by qRT-PCR. The detected expression levels of vtg isoforms in each sample was normalised against that of β-actin, and the data were expressed as a ratio to the corresponding normalised level of each vtg isoform in the liver of untreated male fish. N=3, two-tail t-test, ***p<0.001

supplementary method

supplementary file 1

supplementary file 2

supplementary file 3

supplementary file 4

supplementary file 5

supplementary file 6

## Declaration of interest

The authors declare that there is no conflict of interest that could be perceived as prejudicing the impartiality of the research reported.

## Funding

This work was supported by a general research fund grant (Project No. CityU 160213) from the Research Grants Council (RGC) of the Hong Kong Special Administrative Region, China, to S H C, and a Strategic Research Grant (Project No. CityU 7004661) from City University of Hong Kong to Y W L.
