# Supplementary figures and images for: Estrogen accelerates heart regeneration by promoting the inflammatory response in zebrafish
Source: J Endocrinol. 2020 Jan 24;245(1):39–51. doi: 10.1530/JOE-19-0413 (PMC7040496; doi:10.1530/JOE-19-0413)

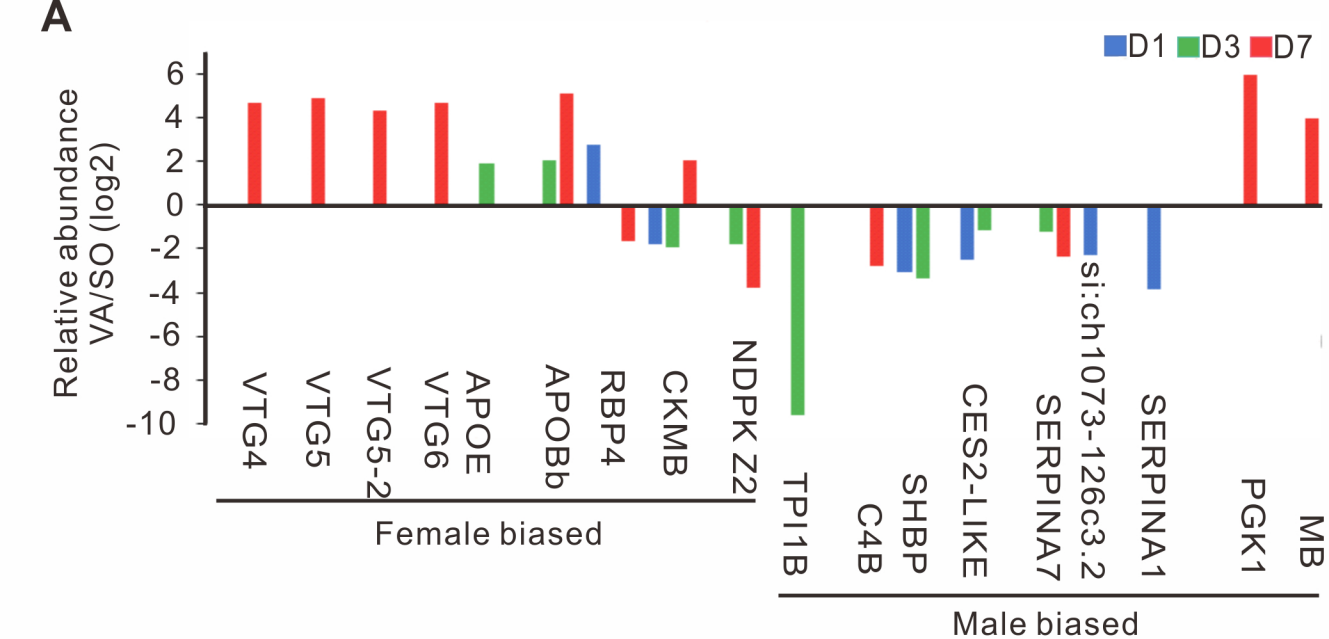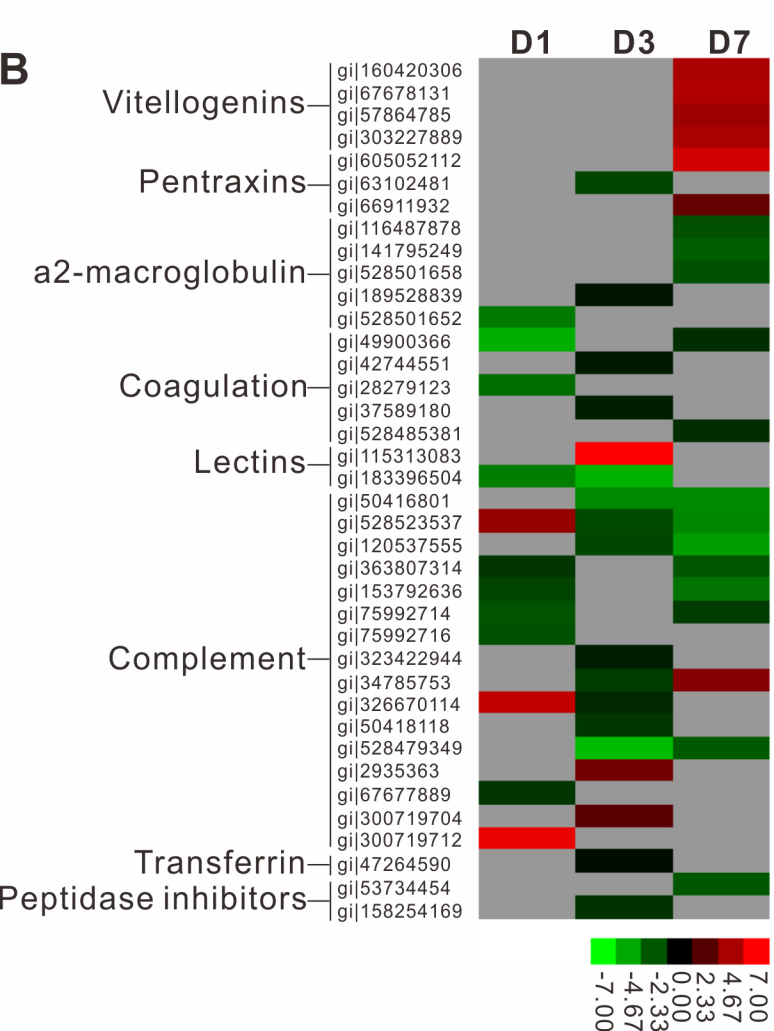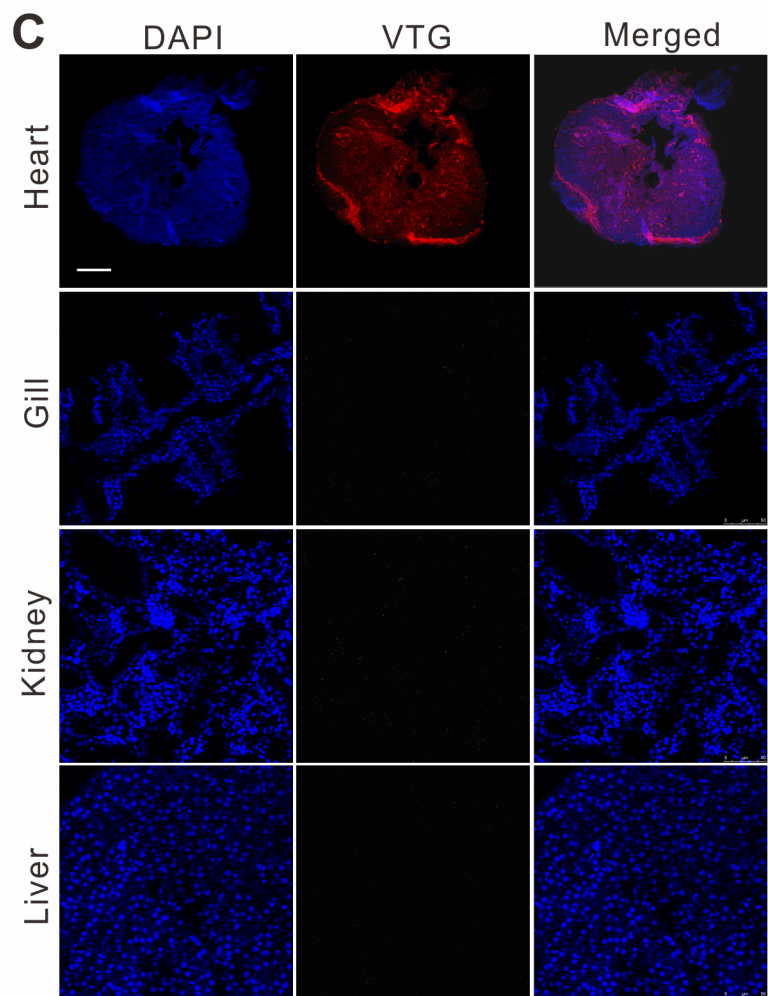

Supplement: Figure S1. Vitellogenin accumulates in the male zebrafish heart after cardiac damage. A. Ratio of the relative abundance of selected plasma proteins on days 1, 3 and 7 post cardiac injury. VA: ventricular amputation, SO: sham operation. N=3-5. B. Relative abundance of known acute response proteins i [file supplementary_figure_1.pdf]

**A**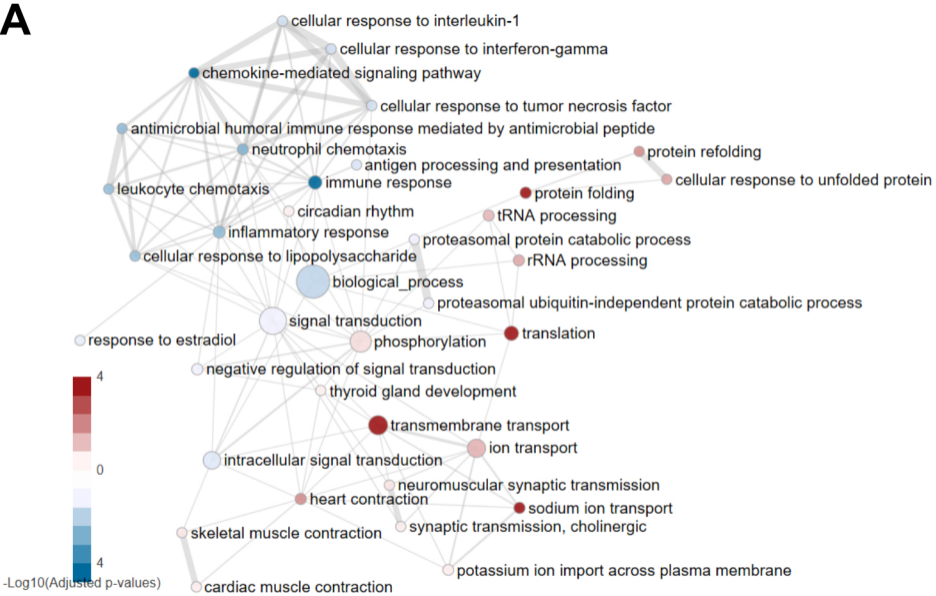**B**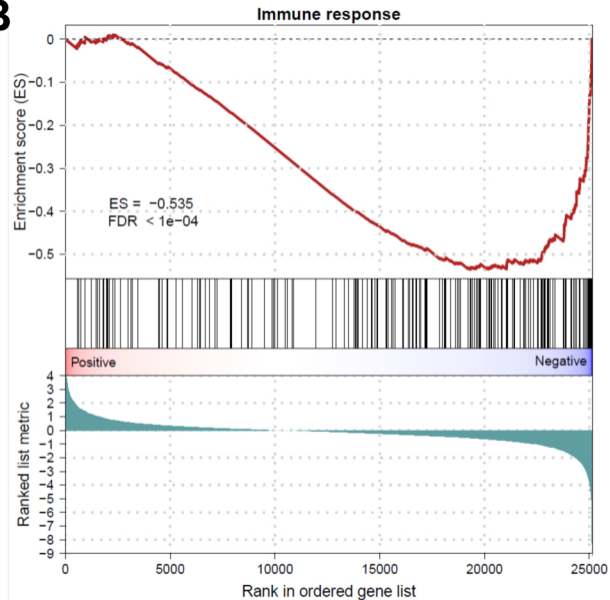

Supplement: Figure S2. Comparative transcriptome analyses between female and male zebrafish 7 days post cardiac injury. A. Enrichment map showing the gene profile in female and male heart at 7 dpc. Red color represents NES>0, and gene set upregulated; blue color represents NES< 0, and gene set downregulated. Th [file supplementary_figure_2.pdf]

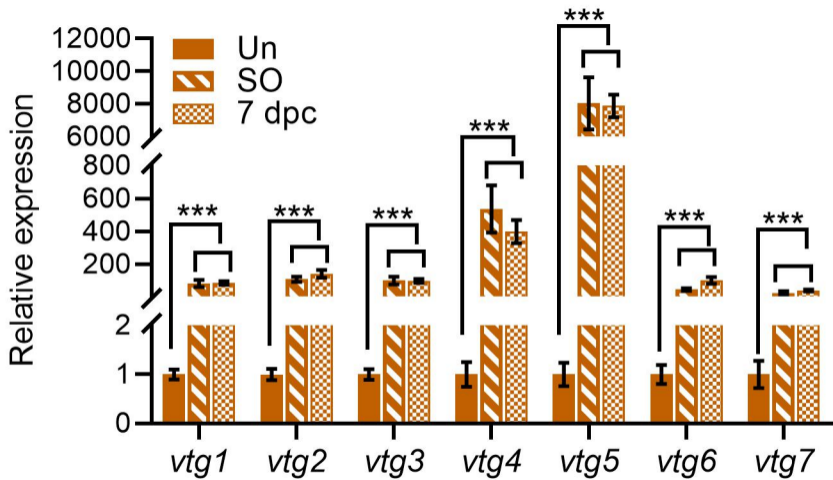

Supplement: Figure S3. Expression of vitellogenin isoforms in the male liver are stimulated by cardiac damage. Expression levels of vtg 1-7 in male zebrafish liver with heart untreated (Un), sham operation (SO) and cryoinjury at 7 dpc, as measured by qRT-PCR. The detected expression levels of vtg isoforms in ea [file supplementary_figure_3.pdf]
