# Supplementary material for: Estrogen accelerates heart regeneration by promoting the inflammatory response in zebrafish
Source: J Endocrinol. 2020 Jan 24;245(1):39–51. doi: 10.1530/JOE-19-0413 (PMC7040496; doi:10.1530/JOE-19-0413)
Supplement: supplementary method [file supplementary_material.pdf]

Supplementary method

Estrogen accelerates heart regeneration by promoting inflammatory responses in zebrafish

Shisan Xu[1], Fangjing Xie[1], Li Tian[1], Samane Fallah[1], Fatemeh Babaei[2], Sinai HC Manno[1], Francis A. M. Manno III [3], Lina Zhu[1], Kin Fung Wong[4], Yimin Liang[2], Rajkumar Ramalingam[2], Lei Sun[4], Xin Wang[1], Robert Plumb[5], Lee Gethings[5], Yun Wah Lam[2]* and Shuk Han Cheng[1,6,7]*

[1] Department of Biomedical Sciences, College of Veterinary Medicine and Life Science, City University of Hong Kong, Hong Kong SAR, PRC.

[2] Departments of Chemistry, City University of Hong Kong, Hong Kong SAR, PRC.

[3] School of Biomedical Engineering, Faculty of Engineering, University of Sydney, Sydney, New South Wales, Australia

[4] Department of Biomedical Engineering, Polytechnic University of Hong Kong, Hong Kong SAR, PRC

[5] Waters Technologies Corporation. 34bMaple Street, Milford, MA 01757 USA

[6] State Key Laboratory of Marine Pollution (SKLMP) at City University of Hong Kong, Hong Kong SAR, PRC.

[7] Department of Materials Science and Engineering, College of Science and Engineering, City University of Hong Kong, Hong Kong SAR, PRC.

*Correspondence: yunwlam@cityu.edu.hk, bhcheng@cityu.edu.hk.

Mass spectrometry

Proteins from plasma collected from individual fish (typically about 20 μg) were precipitated in 1 mL of acetone for 60 min at -20°C and pelleted at 1000g for 10 min at 4 ^{o}C. The pellet was air-dried (30 min) and then dissolved in 8M urea in 10 mM Tris-HCl (pH 8.0). After clarification (10000 g, 10 min, RT), the supernatant was reduced by 10mM dithiothreitol (R0861, Thermo Scientific) in 50 mM ammonium bicarbonate (09830, Sigma-Aldrich; 30 min, RT), and alkylated in 50mM iodoacetamide (90034, Thermo Scientific) in 50 mM ammonium bicarbonate (20 min, RT). LysC (Roche) was then added to the protein mixtures and incubated for 120 min at RT. The samples were then diluted with ammonium bicarbonate (50 mM, pH 8.0) so that the final concentration of urea was 1 M, followed by the addition of 1 μg trypsin (06369880103, Roche). The mixtures were incubated overnight at 37°C and dried in a vacuum centrifuge. Peptides were dissolved in a small amount of 0.1% trifluoroacetic acid (302031, Sigma-Aldrich; TFA), followed by purification with the use of ZipTip (MA01821, Millipore), following manufacturer's instruction.

Mass spectrometry was performed on the Thermo Scientific Q-Exactive Plus Mass Spectrometer (Thermo Fisher Scientific, Bremen, Germany) coupled to a Thermo Ultimate 3000 RSLCnano HPLC system. About the 0.5 ug of the peptide mixture was loaded onto a 250nl OPTI-PAK trap (Optimize Technologies, Oregon City, OR) custom packed with Michrom Magic C18 5 μm solid phase (Michrom Bioresources, Auburn, CA). Chromatography was performed a Thermo EasySpray 25 cm × 75 μm C18 2 μm column, using 0.2 % formic acid in both Solution A (98%water/2% acetonitrile) and Solution B (80% acetonitrile/10% isopropanol/10% water), in a gradient from 2%B to 35%B over 140 min at a flowrate of 325 nL/min. The

Q-Exactive Plus mass spectrometer was set up with a FT survey scan from 340-1500 m/z at resolution 70,000 (at 200m/z), followed by HCD MS/MS scans on the top 15 ions at resolution 17,500. The MS1 AGC target was set to 1e6 and the MS2 target was set to 2e5 with max ion inject times of 50ms and 75ms respectively. Dynamic exclusion placed selected ions on an exclusion list for 30 seconds. Charge exclusion was used to perform MS/MS only on +2, +3, and +4 ions.

MS/MS peak lists were exported as an .mgf file and proteins were identified via automated Mascot database searching (Matrix Science 2.2.04) of all tandem mass spectra against of the Danio rerio Protein Index protein sequence database that contained all zebrafish protein entries (143,725 sequences) from NCBI RefSeq (version 51). The instrument setting for the Mascot search was specified as "ESI-Trap." Parameters used for the database search were as follows: a maximum of two missed cleavages; carbamidomethylation of cysteine as a fixed modification and oxidation of methionine, acetylation of protein N-term and Gln→pyro-Glu conversion as variable modifications; trypsin as the enzyme; a peptide mass tolerance of 10 ppm; a fragment mass tolerance of 0.6 Da; and an ion score of 35 as the cut-off, using a significance threshold of $p < 0.05$. After peptide identification, any peptides that had conflicting assignments were resolved, either one of identical proteins or by assignment to proteins with the largest number of peptides already present (by following Occam's Razor principle).

The acquired MS data (in Thermo ".raw" format) from 3 biological replicates in the SO and Untreated groups (i.e., three separate plasma samples each collected from one individual fish) and 4 biological replicates in the VA group were quantitated by using

Progenesis LC-MS (version 2.5, Nonlinear) as previously described (Babaei et al. 2013).
